# Spn1 Maintains Nucleosome Organization at Transcription End Sites

**DOI:** 10.3390/epigenomes10040061

**Published:** 2026-09-30

**Authors:** Uwe Schwartz

**Affiliations:** NGS Analysis Center Biology and Preclinical Medicine, University of Regensburg, D-93053 Regensburg, Germany; uwe.schwartz@ur.de; Tel.: +49-941-943-2867

**Keywords:** Spn1, transcription, nucleosomes, termination, Spt6

## Abstract

Background/Objectives: Transcription by RNA polymerase II (RNAPII) occurs within chromatin and requires continuous disassembly and reassembly of nucleosomes. Histone chaperones associate with RNAPII, facilitate transcription, and maintain nucleosome organization. This study investigated the role of the conserved histone chaperone Spn1 in coordinating chromatin organization with transcription termination in *Saccharomyces cerevisiae*. Methods: Spn1 interacts with elongating RNAPII via Spt6 and its function was examined by reanalyzing published MNase-seq, RNAPII ChIP-seq, NET-seq, and TSS-seq datasets. Results: Mutations that disrupt the Spn1–Spt6 interaction at nonpermissive temperatures caused a pronounced loss of nucleosome array regularity at polyadenylation (pA) sites, whereas nucleosome organization around transcription start sites was less affected. These mutants also showed shorter nucleosome repeat lengths at gene ends. RNAPII accumulated near pA sites and displayed increased termination-proximal stalling, consistent with impaired transcription termination. In addition, transcription of neighboring downstream genes in tandem orientation was preferentially reduced, suggesting interference between termination of the upstream gene and initiation at the neighboring promoter. Conclusions: Together, these results identify Spn1 as an important determinant of chromatin organization at gene ends and suggest that it helps maintain transcriptional fidelity across closely spaced genes.

## 1. Introduction

Transcription is a fundamental and tightly regulated cellular process in which genetic information is copied from DNA into RNA. In eukaryotes, transcription occurs within the context of chromatin [1]. The fundamental unit of chromatin is the nucleosome, which consists of 147 bp of DNA wrapped around an octamer of core histone proteins [2]. Packaging of DNA into nucleosomes creates dynamic barriers to transcription that must be navigated by the transcriptional machinery [3]. Histone chaperones and chromatin remodelers play crucial roles in modifying chromatin structure to either facilitate or impede transcription [3,4]. During transcription, nucleosomes undergo continuous cycles of disassembly and reassembly, allowing RNAPII to traverse chromatin while maintaining nucleosome organization [5]. Although the roles of chromatin in transcription initiation and elongation are well studied, its contribution to transcription termination remains less well understood. Proper transcription termination is critical for defining gene boundaries, ensuring efficient 3′-end processing of nascent RNAPII transcripts, and maintaining overall transcriptional fidelity [6]. Failure to terminate transcription correctly can result in RNAPII transcribing into downstream genomic regions, potentially disrupting neighboring gene expression and producing aberrant, unstable transcripts.

Multiple histone chaperones, including Spt6, Spn1, and the FACT complex (Spt16 and Pob3), associate with elongating RNAPII [7,8]. Depletion of Spt6 reduces productive elongation and causes transcription termination defects, resulting in readthrough transcripts downstream of the polyadenylation (pA) site [9]. Furthermore, loss of Spt6 function disrupts nucleosome organization, alters histone modifications such as H3K36 methylation, and causes widespread transcriptional defects, including cryptic initiation and aberrant antisense transcription [10,11,12]. Spt6 is evolutionarily conserved and interacts through multiple domains with transcription elongation factors, including DSIF (Spt4 and Spt5), the polymerase-associated factor 1 complex (Paf1C), TFIIS, FACT, and Spn1. These interactions stimulate RNAPII elongation through chromatin and promote nucleosome reassembly behind the polymerase [7,13].

The essential histone chaperone Spn1 forms a stable interaction with Spt6 and is a core component of the RNAPII elongation complex [13,14]. Temperature-sensitive single-amino-acid mutations in Spn1 or Spt6 that disrupt the Spn1–Spt6 interaction cause defects in nucleosome positioning and spacing downstream of the transcription start sites (TSS) of expressed genes and induce intragenic and antisense transcripts at nonpermissive temperatures [8,15]. Although the roles of Spt6 and Spn1 in transcription elongation and nucleosome reassembly are well documented, their precise contributions to transcription termination, particularly in the context of nucleosome organization at pA sites, remain to be defined.

Here, the contribution of the Spn1–Spt6 interaction to nucleosome organization at the 3′ ends of genes and to transcription termination in *Saccharomyces cerevisiae* was investigated. MNase-seq, RNAPII ChIP-seq, NET-seq, TSS-seq, and ChIP-exo datasets were integrated to compare chromatin organization at transcription start and pA sites and to assess its relationship with RNAPII gene transcription. Disruption of the Spn1–Spt6 interaction was found to preferentially impair nucleosome array organization at pA sites and to be associated with increased termination-proximal RNAPII stalling and reduced transcription initiation at neighboring downstream genes in tandem orientation. These findings suggest that chromatin organization at gene ends is maintained by Spn1 and that transcriptional fidelity at closely spaced gene boundaries is thereby supported.

## 2. Results

### 2.1. Spn1 Maintains Nucleosome Array Organization at pA Sites

To investigate the function of the histone chaperone Spn1 during transcription, recently published MNase-seq datasets containing single-amino-acid mutations in either Spn1 or Spt6 were reanalyzed using the comprehensive MNase-seq analysis pipeline nucDetective [15,16]. These mutations are located at the Spn1–Spt6 interaction interface and were shown in vitro to disrupt heterodimer formation [14]. Checking the consistency of replicates after running the Profiler workflow of nucDetective, two samples appeared to have been mislabeled and exchanged between the *spn1-F267E* and *spn1-R263D* groups. This was confirmed by examining alignment mismatches to the reference genome, which clearly identified the genotype of each sample (Figure S1A). After correcting the sample labels, correlation analysis of the nucleosome profiles revealed two major groups: wild type (WT) and *spn1-R263D* samples clustered together and were distinct from *spt6-F249K* and *spn1-F267E* samples (Figure S1B). This clustering agrees well with in vivo growth assays showing growth defects for *spt6-F249K* and *spn1-F267E* at nonpermissive temperatures, whereas *spn1-R263D* grows normally [14]. Consistent with recent studies, genome-wide nucleosome spacing was, on average, wider in mutants with reduced Spn1–Spt6 binding at nonpermissive temperatures. This effect was particularly evident in the nucleosome array downstream of the +1 nucleosome at the TSS, with the exception of *spn1-R263D*, which closely resembled WT (Figure S1C,D) [8,15].

**Figure 1:**
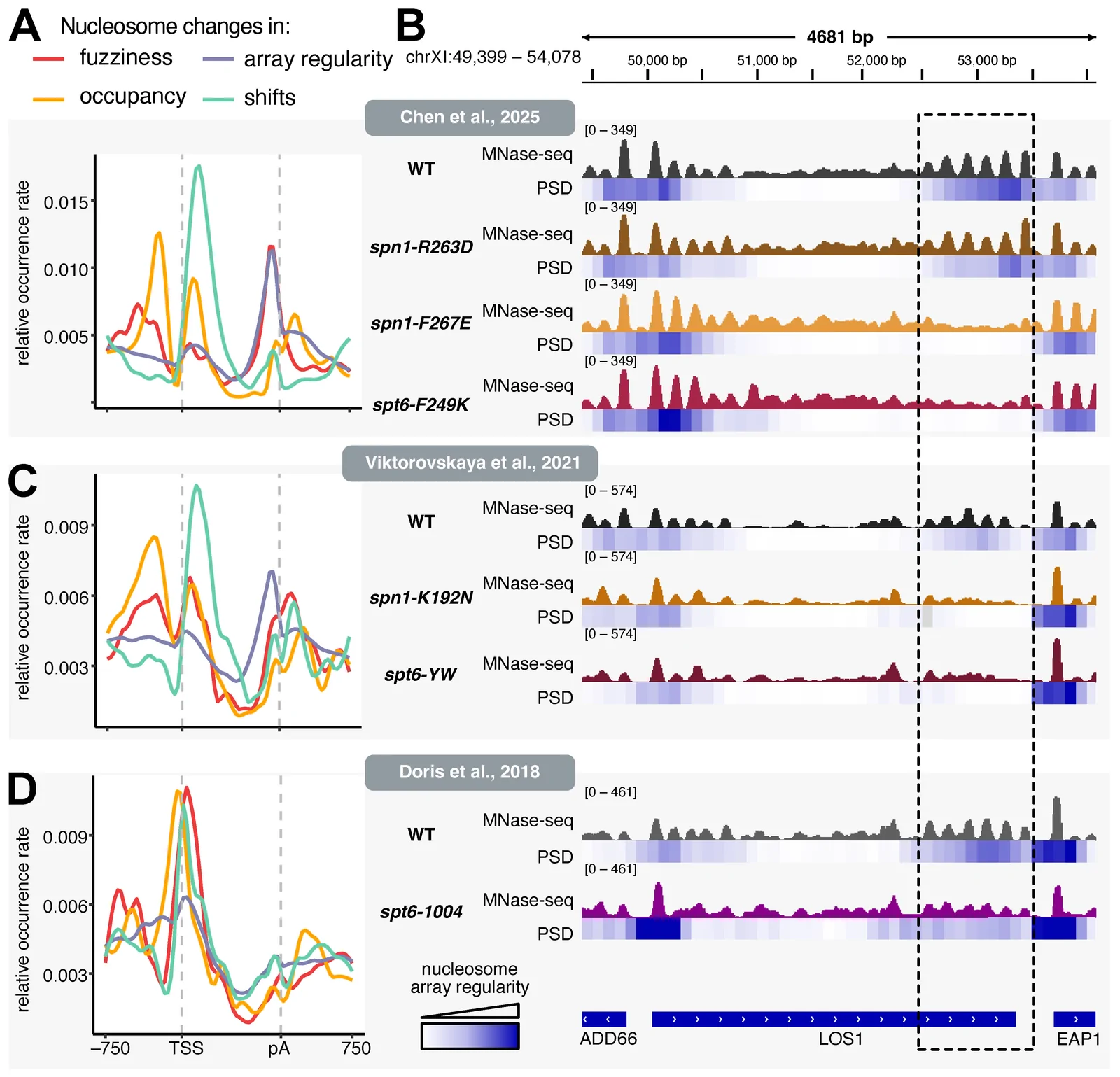
Spn1 maintains nucleosome array organization at transcription end sites: (**A**) Distribution of nucleosome organization changes in Spn1–Spt6 interaction mutants. Nucleosomes showing large changes in fuzziness (red), occupancy (orange), positioning (turquoise), or array regularity (purple) across spn1/spt6 mutants taken from [15] were identified using the nucDetective pipeline. The distributions of the dynamic nucleosome features were assessed over all genes, which have been scaled to the same length. Relative occurrence rates were calculated as the number of occurrences of each dynamic nucleosome feature at a given relative position divided by the total number of occurrences of that feature. (**B**) Genome browser snapshot showing nucleosome array regularity at LOS1. MNase-seq profiles of mono-nucleosomes and power spectral densities (PSD) were generated using the nucDetective pipeline. PSD signal at the approximate nucleosome repeat length (here 155 bp) was used as a measure of nucleosome array regularity. MNase-seq track colors distinguish the indicated WT and mutant strains. The dashed frame highlights the region at the 3′ end of LOS1 used to illustrate changes in nucleosome array regularity. (**C**) As in (**A**), except that MNase-seq data for spn1/spt6 mutants and WT were obtained from [8]. (**D**) As in (**A**), except that MNase-seq data of the spt6-1004 and WT were obtained from [12].

Recent studies of Spt6 and Spn1 in chromatin organization have focused primarily on the first nucleosomes downstream of the TSS [8,12,15]. The Inspector workflow of nucDetective enables a less biased analysis by ranking genome-wide nucleosome arrangements according to the magnitude of change [16]. Multiple nucleosome features, including occupancy, fuzziness, positioning, and array regularity, can therefore be analyzed simultaneously across genotypes. Mapping the nucleosomes with the largest changes in occupancy or position (shifts) between mutants and WT revealed an enrichment near the TSS (Figure 1A). Occupancy changes were most prominent at the −1 and +1 nucleosomes, whereas positional shifts affected the first nucleosomes downstream of the TSS. In contrast, changes in nucleosome array regularity and fuzziness were more prominent at pA sites (Figure 1A,B). Analysis of an independent MNase-seq dataset containing two additional mutants with impaired Spt6-Spn1 binding (*spn1-K192N* and *spt6-YW*), both of which exhibit growth defects at nonpermissive temperatures, yielded a very similar pattern, supporting the robustness of the analysis (Figure 1C) [8]. By contrast, the *spt6-1004* mutant, in which Spt6 protein levels are markedly reduced at nonpermissive temperatures, showed the strongest disruption of nucleosome organization at the TSS (Figure 1B,D). This suggests that the loss of nucleosome array regularity at pA sites cannot be attributed to reduced Spt6 function alone and may be particularly sensitive to disruption of the Spt6-Spn1 interaction [8,12].

**Figure 2:**
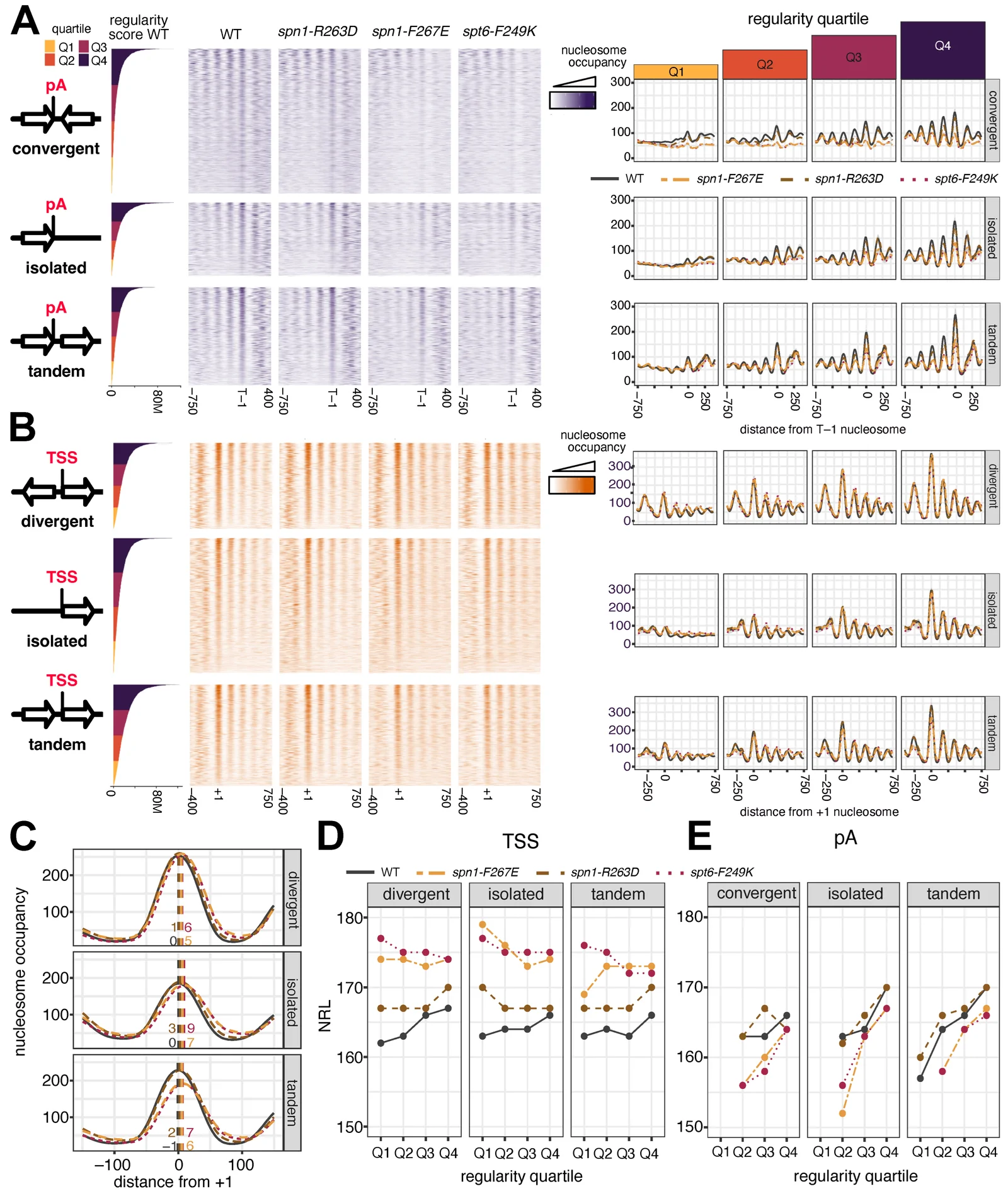
Nucleosome array regularity at the transcription end sites is more affected in Spn1–Spt6 interaction mutants than at the transcription start sites. (**A**) Nucleosome occupancy centered on the terminal nucleosome (T-1) adjacent to the polyadenylation site (pA). Heatmaps show nucleosome occupancy in WT, *spn1-R263D*, *spn1-F267E* and *spt6-F249K* strains, displayed separately for convergent, isolated, and tandem genes. Genes are sorted by the mean WT nucleosome array regularity score within the 300 bp region upstream of the T-1 nucleosome (bar plot, left) and divided into quartiles Q1–Q4, indicated by the corresponding color coding. Metaplots (right) show the corresponding mean nucleosome occupancy for each strain and are additionally stratified into quartiles (Q1–Q4) according to the nucleosome array regularity score. The grey ribbon indicates the standard error of the mean (*n* = 3). (**B**) As in (**A**), except that profiles are centered on the +1 nucleosome downstream of the TSS and genes are grouped as divergent, isolated, or tandem. (**C**) Details of +1 nucleosome positioning in WT, *spn1-R263D*, *spn1-F267E*, and *spt6-F249K* strains. +1 nucleosome positions are shown separately for divergent, isolated, and tandem genes and are aligned to the +1 nucleosome dyad position determined in the WT strain. Dashed vertical lines indicate the nucleosome dyad positions, determined from the maxima of LOESS-smoothed nucleosome occupancy profiles. Labels show the corresponding positions relative to the WT +1 nucleosome. (**D**) Nucleosome repeat length (NRL) downstream of the TSS in WT, *spn1-R263D*, *spn1-F267E* and *spt6-F249K* strains. Genes were grouped by gene arrangement into divergent, isolated, and tandem classes and stratified by nucleosome array regularity into quartiles (Q1–Q4). Dyad positions of the first four nucleosomes were defined from maxima of LOESS-smoothed occupancy profiles, and the NRL was estimated as the slope of a linear fit through consecutive nucleosome positions. Only linear fits with NRL ≥ 150 bp and R^2^ > 0.99 are shown. Points indicate NRL estimates for each regularity quartile. (**E**) As in (**D**), except that the NRL was calculated from the last four nucleosomes upstream of the pA site and genes were grouped as convergent, isolated, or tandem genes.

To determine which genes were most affected, all protein-coding genes were grouped according to the distance and orientation of the nearest gene downstream of the pA site. For each gene, the nucleosome closest to the annotated pA site was used as a reference and termed the termination-1 (T-1) nucleosome (Supplemental Table S1), analogous to the use of the +1 nucleosome as a reference at the TSS [17,18]. The distance from the T-1 nucleosome to the nearest downstream gene was then calculated. Genes with no neighboring protein-coding gene within 500 bp were classified as isolated, whereas genes with a neighboring gene within 500 bp were classified as convergent or tandem according to the orientation of the downstream gene (Supplemental Table S1). Within each group, genes were sorted by nucleosome array regularity at the pA site and divided into quartiles (Figure 2A). Gene length distributions were then assessed across groups (Figure S2A). At isolated pA sites, the nucleosome array regularity was largely independent of gene length, whereas regularity at convergent and tandem pA sites correlated with gene length. At convergent genes, the highest regularity (Q4) was observed for relatively short genes (median 702 bp), corresponding to fewer than five nucleosomes within the gene body. Well-defined nucleosome arrays can arise through statistical positioning, in which a barrier, such as the +1 nucleosome, constrains the positions of adjacent nucleosomes, producing an ordered array whose positional regularity decays with increasing distance from the barrier [17,19]. The enrichment of short genes in Q4 therefore suggests that nucleosome regularity at the pA sites of convergent genes is largely driven by statistical positioning from the +1 nucleosome. In contrast, nucleosome regularity at the pA sites of tandem genes was lowest for short genes and increased with gene length. Immediately downstream of the T-1 nucleosome lies the nucleosome-depleted region of the next gene, where assembly of the transcription initiation machinery creates a barrier to nucleosome movement and can statistically position nucleosomes upstream of the T-1 nucleosome [17]. The enrichment of longer genes among tandem pA sites with high regularity suggests that, in short genes, statistical nucleosome positioning from the +1 nucleosome may compete with positioning imposed by the downstream promoter at the T-1 nucleosome.

Consistent with the TSS-centered analysis (Figure S1D) nucleosome profiles of the *spn1-R263D* at the pA sites closely resembled WT, whereas the *spn1-F267E* and *spt6-F249K* showed a global loss of nucleosome array regularity across all gene groups (Figure 2A). The strongest effect was observed at convergent pA sites, where nucleosome regularity at 3′ ends of genes was almost completely lost for approximately 75% of genes. Tandem genes showed the highest nucleosome array regularity at pA sites in WT. In this configuration, the position of the T-1 nucleosome remained relatively robust even in the *spn1-F267E* and *spt6-F249K* mutants. In contrast to isolated and convergent pA sites, the nucleosome immediately downstream of the T-1, which corresponds to the +1 nucleosome of the neighboring downstream gene in tandem configurations, also remained well positioned and showed only minor changes relative to WT.

Nucleosome array regularity was analyzed analogously at the TSS (Figure 2B). Nucleosome positioning at the TSS was less affected in *spn1-F267E* and *spt6-F249K* mutants, particularly in quartile 4 (Q4), which has the highest array regularity. Formation of the +1 nucleosome was relatively robust and was detectably reduced only at tandem TSSs with high array regularity (Figure 2B). Nevertheless, detailed analysis of +1 nucleosome positioning revealed an average downstream shift of up to 9 bp in *spn1-F267E* and *spt6-F249K* mutants (Figure 2C). The most pronounced change was an increased nucleosome repeat length (NRL) downstream of the +1 nucleosome, particularly in genes with lower array regularity at the TSS (Figure 2B,D). By contrast, nucleosome spacing at the pA sites was affected in the opposite direction, with shorter NRLs in *spn1-F267E* and *spt6-F249K* mutants (Figure 2E).

The changes in nucleosome array regularity at pA sites or TSSs observed in *spn1-F267E* and *spt6-F249K* at nonpermissive temperatures were independently reproduced using the *spn1-K192N* and *spt6-YW* mutants (Figure S3A,B). In addition, the *spt5-QS* suppressor mutation largely reversed the nucleosome positioning defects caused by *spt6-YW* at the TSS and, to a lesser extent, at pA sites in *spt6-YW spt5-QS* double-mutant strains (Figure S3A,B) [8]. At the permissive temperature, nucleosome positioning was less affected at both TSSs and pA sites (Figure S3C,D). Nevertheless, loss of nucleosome array regularity was already clearly detectable at the pA sites at 30 °C, whereas the TSS was comparatively preserved.

The *spt6-1004* mutant, which reduces Spt6 protein levels associated with RNAPII [12], showed a more severe loss of array regularity at both TSSs and pA sites (Figure S3E,F), suggesting a distinct role for Spt6 in nucleosome organization at the TSS that is at least partly independent of Spn1. Together, these analyses show that mutations disrupting the Spn1–Spt6 interaction preferentially impair nucleosome array regularity at pA sites, supporting a requirement for Spn1 in maintaining nucleosome organization at the 3′ ends of genes.

### 2.2. Spn1 Regulates RNAPII Processivity and Termination

**Figure 3:**
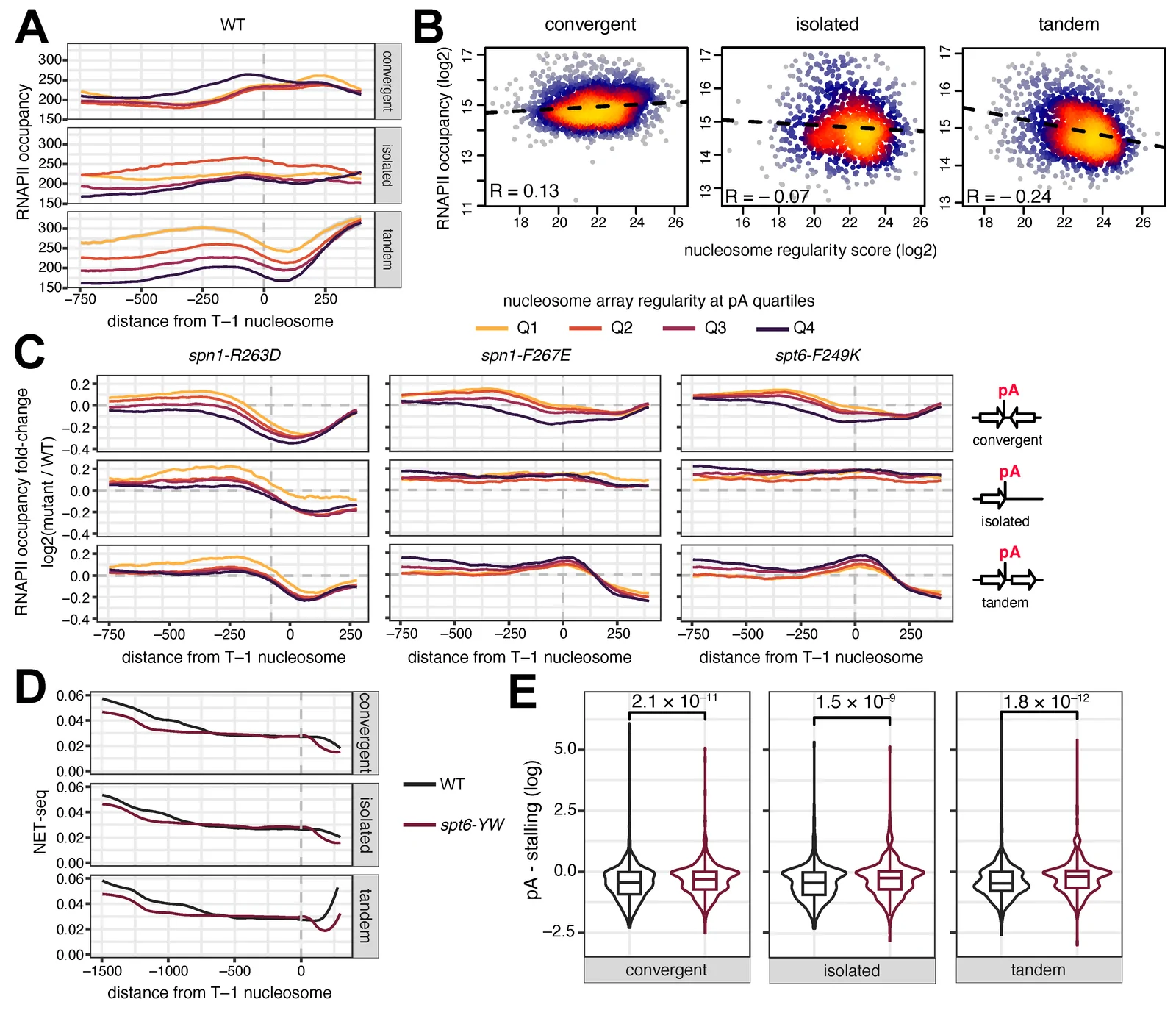
Impaired Spn1–Spt6 interaction alters RNAPII occupancy and increases termination-proximal stalling. (**A**) RNAPII ChIP-seq profiles centered on the T-1 nucleosome in WT cells at 37 °C. Profiles are shown separately for convergent, isolated and tandem genes and were stratified into quartiles (Q1–Q4) according to nucleosome array regularity at the pA site as indicated by the color code. (**B**) Correlation between nucleosome array regularity and RNAPII occupancy at the pA site in WT cells. RNAPII occupancy was quantified as the summed ChIP-seq signal within −250 to +250 bp of the T-1 nucleosome. Dashed lines indicate linear regression fits, and the Spearman correlation coefficients (R) are shown in each panel. Point density is indicated by the color scale, with yellow colors representing regions of higher point density and grey colors lower point density. (**C**) Changes in RNAPII occupancy in Spn1–Spt6 interaction mutants relative to WT at 37 °C. Meta-gene profiles are shown separately for convergent, isolated, and tandem genes and stratified into nucleosome array regularity quartiles (Q1–Q4). (**D**) Strand-specific NET-seq profiles of transcriptionally engaged RNAPII in WT (black) and *spt6-YW* (purple) cells at 37 °C. The analysis was restricted to genes longer than 1.5 kb and to NET-seq reads in the sense orientation. Median profiles are shown for convergent, isolated and tandem genes. (**E**) pA-proximal RNAPII stalling is increased in *spt6-YW* cells relative to WT at 37 °C. The pA-stalling index was calculated for each gene as the log2 ratio of the median NET-seq signal surrounding the T-1 nucleosome (−100 to +100 bp) to the median signal in the upstream gene body (−1250 to −750 bp relative to T-1 nucleosome). pA-stalling indices were compared between WT and *spt6-YW* cells using a paired, two-sided *t*-test. Exact *p*-values are indicated above the plots.

To determine how nucleosome array regularity at pA sites and its disruption in Spn1–Spt6 interaction mutants relate to active transcription, RNAPII ChIP-seq and NET-seq data were analyzed. At tandem pA sites, RNAPII occupancy within −250 to +250 bp of the T-1 nucleosome was negatively correlated with nucleosome array regularity, indicating higher transcriptional activity at less regularly positioned nucleosomes (Figure 3A,B), potentially as a consequence of frequent nucleosome dis- and reassembly during active transcription [20]. Correlations were weak or negligible at convergent or isolated pA sites in WT. The relatively high RNAPII abundance upstream of convergent pA sites with high nucleosome regularity (Q4) can likely be explained by the enrichment of short genes in this group, for which the TSS lies close to the pA site (Figure S2). WT-NET-seq profiles at nonpermissive temperatures closely resembled RNAPII ChIP-seq occupancy profiles (Figure 3A and Figure S4A). To distinguish initiating from elongating RNAPII, published ChIP-exo data targeting Ser2-phosphorylated RNAPII C-terminal domain (CTDSer2; elongation) or Ser5-phosphorylated CTD (CTDSer5; initiation) under standard growth conditions were analyzed (Figure S4B) [21]. Because of the compact gene organization, the CTDSer2 signal from an upstream gene overlaps with the CTDSer5 signal at the downstream promoter in tandem genes and, to a lesser extent, in isolated genes with high nucleosome array regularity (Q4). At convergent genes, CTDSer2 signals from neighboring genes overlap around the pA sites. CTDSer2 occupancy showed mild inverse correlations with nucleosome array regularity in all gene groups (Figure S4C), consistent with reduced array regularity at higher RNAPII occupancy. Spn1 and Spt6 occupancy peaked directly upstream of pA sites and showed inverse correlations with nucleosome regularity of a similar magnitude to CTDSer2 (Figure S4B,C).

To assess the role of Spn1 in transcriptional regulation at the 3′ ends of genes, the RNAPII occupancy in *spn1-R263D*, *spn1-F267E* and *spt6-F249K* mutants was compared with WT at nonpermissive temperatures (Figure 3C). RNAPII occupancy tended to be higher upstream of the pA site and lower downstream in Spn1–Spt6 interaction mutants, with the exception of isolated pA sites in *spn1-F267E* and *spt6-F249K*. However, the local distribution of RNAPII around the pA site differed among gene configurations. At the T-1 nucleosome, RNAPII abundance was not markedly altered at convergent genes, whereas the greatest changes were observed at tandem genes in *spn1-F267E* and *spt6-F249K* mutants. RNAPII accumulation at the T-1 nucleosome was not observed in *spn1-R263D*, in which nucleosome regularity at the pA sites also remained largely unchanged (Figure 2A).

Increased RNAPII occupancy can reflect either increased transcription or reduced polymerase progression and local stalling. To distinguish between these possibilities, nascent transcription was analyzed by NET-seq in long genes (>1.5 kb) in WT and *spt6-YW* cells (Figure 3D) [8]. In *spt6-YW* mutants, fewer transcriptionally engaged polymerases were detected more than 750 bp upstream of the T-1 nucleosome, consistent with recent reports of reduced processivity after rapid Spn1 depletion in human cell lines [22]. In contrast, NET-seq signal at the T-1 nucleosome was similar between WT and *spt6-YW*, indicating increased relative RNAPII stalling at the 3′ ends of genes in the mutant. This was confirmed by calculating a pA-proximal stalling index for each gene (Figure 3E). The median NET-seq signal around the T-1 nucleosome (−100 to +100 bp) was divided by the median signal in the upstream gene body (−1250 to −750 bp), revealing significantly higher stalling in *spt6-YW*. Immediately downstream of the T-1 nucleosome, however, the abundance of transcriptionally engaged RNAPII dropped sharply in *spt6-YW*. This may reflect an upstream shift in polyadenylation and termination, consistent with the premature use of upstream pA sites reported after loss of Spt6 [23].

Together, these results indicate that impaired Spn1–Spt6 interaction alters RNAPII processivity and increases termination-proximal stalling at pA sites.

### 2.3. Spn1 Is Required for Initiation Fidelity at Neighboring Genes in Tandem Orientation

**Figure 4:**
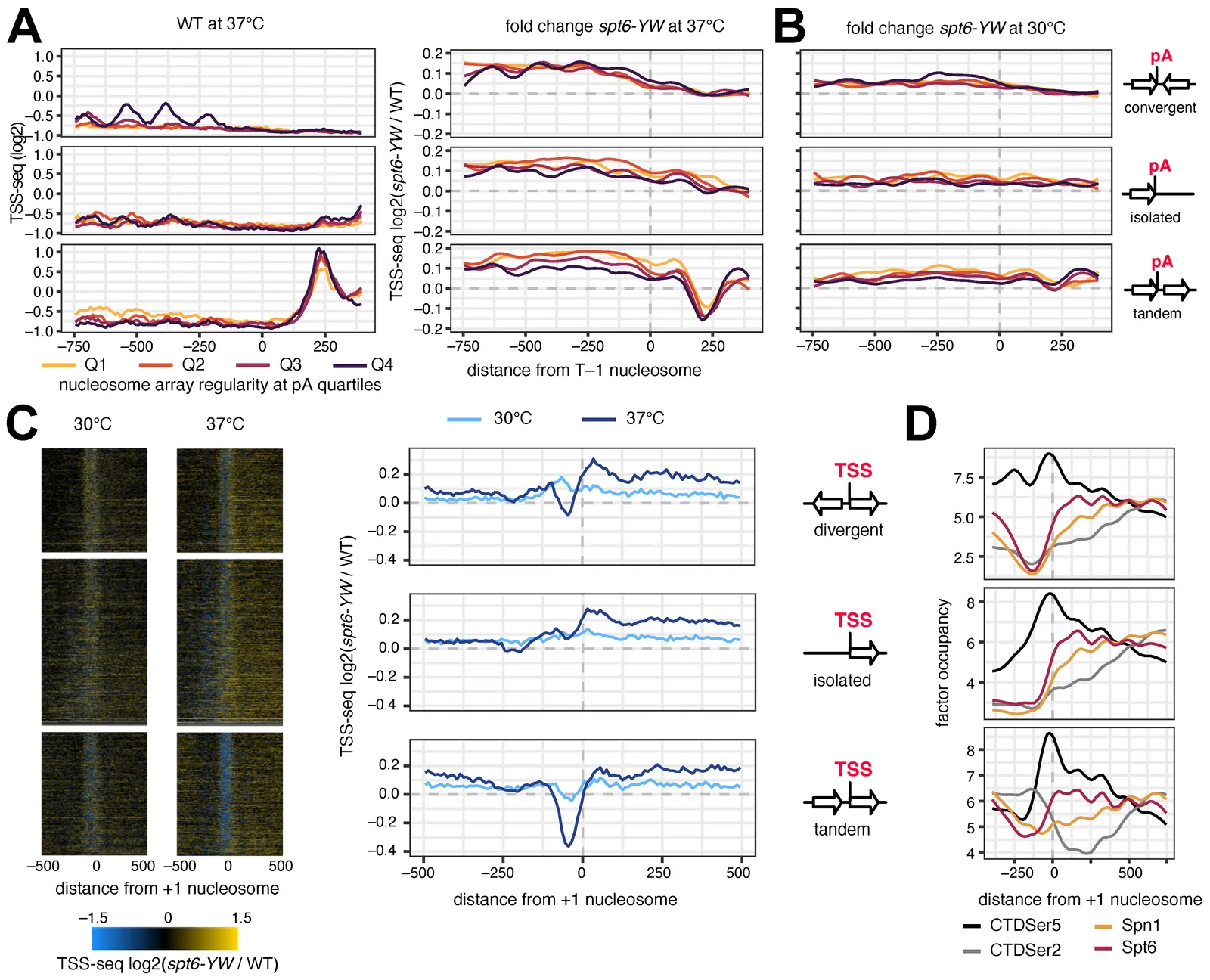
Spn1–Spt6 interaction mutants affect transcription initiation at neighboring genes in tandem orientation. (**A**) Library-size normalized TSS-seq profiles centered on the T-1 nucleosome. The left panel shows TSS-seq signal in WT cells at 37 °C on a log2 scale after addition of a pseudocount of 0.5. The right panel shows log2 fold changes in TSS-seq signal in *spt6-YW* cells relative to WT at 37 °C. Meta-gene plots show the average of convergent, isolated, and tandem genes and are stratified into quartiles (Q1–Q4) according to nucleosome array regularity at the pA site. (**B**) As in (**A**), except that log2 fold-changes are shown at the permissive temperature of 30 °C. (**C**) Changes in transcription initiation centered on the +1 nucleosome in *spt6-YW* relative to WT. Heatmaps show log2 fold changes at permissive and nonpermissive temperatures, as indicated. Corresponding meta-gene profiles are shown on the right. Genes were grouped as divergent, isolated, or tandem. (**D**) ChIP-exo occupancy profiles of initiating RNAPII (CTDSer5), elongating RNAPII (CTDSer2), Spn1, and Spt6 centered on the +1 nucleosome. Profiles are shown separately for divergent, isolated, and tandem gene arrangements, illustrating the spatial overlap between transcription initiation and terminating RNAPII at neighboring tandem genes.

Because impaired Spn1–Spt6 interactions increase cryptic transcription at nonpermissive temperatures, TSS-seq data from the *spt6-YW* mutant were analyzed in relation to nucleosome array regularity at pA sites [8]. In WT, intragenic TSS-seq signal showed little dependence on nucleosome array regularity, except upstream of the T-1 nucleosome at convergent genes with high array regularity (Q4) (Figure 4A). Because this group contains a high proportion of genes shorter than 750 bp, much of the increased signal can be attributed to canonical TSSs (Figure S2). Nevertheless, intragenic transcription initiation upstream of the pA sites was elevated in *spt6-YW* specifically at nonpermissive temperatures (Figure 4A,B), consistent with previous observations [8]. Intragenic initiation was even more pronounced in *spt6-1004* mutants (Figure S5A,B) [12]. However, the degree of nucleosome array regularity did not correlate with the increase in intragenic transcription initiation in either spt6 mutant (Figure 4A,B and Figure S5A,B).

At tandem pA sites a prominent TSS-seq peak appeared approximately 250 bp downstream of the T-1 nucleosome in WT, marking the TSS of the neighboring downstream gene (Figure 4A). Compared with WT, *spt6-YW* showed a pronounced reduction in transcription initiation at these neighboring tandem genes at nonpermissive temperature, an effect that was not observed at the permissive temperature (Figure 4A,B). A similar reduction in neighboring-gene transcription was observed in spt6-1004 mutants (Figure S5A,B). Centering TSS-seq data on the TSSs of all genes confirmed that the effect in *spt6-YW* was strongest for genes in tandem configuration (Figure 4C), whereas initiation at isolated and divergent TSSs showed no comparable global perturbation. By contrast, *spt6-1004* caused broad downregulation of transcription initiation across gene classes, as previously reported (Figure S5C) [12], together with increased cryptic initiation within gene bodies. These findings suggest that impaired termination of the upstream gene in *spt6-YW* may interfere with efficient transcription initiation at the neighboring downstream promoter. To illustrate the close spatial relationship between termination and initiation, ChIP-exo profiles of Spt6, Spn1, initiating RNAPII (CTDSer5), and elongating RNAPII (CTDSer2) were plotted around the TSS (Figure 4D) [21]. At tandem genes, CTDSer5 profiles at the downstream promoter overlap with CTDSer2 profiles from the upstream gene highlighting the requirement for efficient termination to permit transcription initiation at the neighboring gene.

## 3. Discussion

This study identifies Spn1 as a critical determinant of nucleosome array organization, particularly at the pA sites. Whereas previous studies have focused largely on the roles of Spt6 and Spn1 in chromatin organization at the 5′ ends of genes [8,12,15,24], reanalysis of MNase-seq datasets revealed that disruption of the Spt6-Spn1 interface causes a global loss of nucleosome array regularity at the 3′ gene ends. At the TSS, these findings are consistent with previous reports that disruption of the Spt6-Spn1 interaction increases NRL [8,15]. One study proposed that loss of the interaction causes a progressive downstream shift in nucleosomes across gene bodies [8], whereas another reported regular 10.5 bp shifts consistent with rotational realignment of the histone octamer rather than a general spacing defect at actively transcribed genes [15]. In contrast, we observed shorter NRLs at 3′ gene ends in Spn1–Spt6 interaction mutants, indicating that nucleosome rearrangement is context-dependent and may involve a distinct interplay of chromatin remodelers and histone chaperones at TSSs and pA sites. Loss of nucleosome array regularity was also more pronounced at the pA sites and depended on the orientation and distance of the neighboring gene. Convergent genes showed the strongest loss of array regularity, whereas tandem genes were less affected. At convergent genes, the short intergenic region requires coordinated termination and release of two oppositely transcribing RNAPII complexes to minimize transcriptional conflicts. Defective termination could therefore directly interfere with transcription and termination of the opposing neighboring gene. Nucleosomes positioned upstream of pA sites can facilitate termination by slowing RNAPII [25,26], and histone depletion causes a downstream shift in pA site usage [23]. Thus, loss of nucleosome organization near 3′ gene ends in Spt6-Spn1 interaction mutants could contribute to termination defects, with particularly strong consequences at convergent genes that depend on efficient termination of both neighboring transcription units. Recent structural and biochemical analyses support a conserved role of IWS1, the human ortholog of Spn1, in transcription, showing that it interacts with RNAPII, SPT6 and other elongation factors and stimulates RNAPII progression on chromatin [22,27]. However, whether IWS1 also contributes to maintaining nucleosome organization at 3′ gene ends in mammalian cells remains unknown. Consistent with a function in transcription termination in mammalian cells, depletion of SPT6, which recruits IWS1 to RNAPII, causes termination defects and readthrough transcription of both protein-coding and noncoding genes [9,28,29]. Depletion of IWS1, the human ortholog of Spn1, alone was not sufficient to increase readthrough transcription [29]. In yeast, by contrast, Spt6 depletion causes an upstream shift in pA site usage as a consequence of reduced RNAPII elongation rate [23]. In addition, *spt6-YW* strains show increased association of the termination factors Rai1 and Rat1 [8], which promote RNAPII release downstream of pA sites [30,31]. The RNAPII ChIP-seq and NET-seq analyses revealed RNAPII accumulation and increased stalling near transcript ends, followed by a premature decrease in NET-seq signal downstream of the T-1 nucleosome, consistent with an upstream shift in termination. Although human IWS1 appears to be less critical for termination in metazoans [29], maintenance of nucleosome architecture at the pA sites by Spn1 may be particularly important in yeast because of its high gene density and short intergenic regions.

A second major finding was the preferential downregulation of genes in tandem orientation. The most likely explanation is that defective termination of the upstream gene interferes with transcription initiation at the neighboring downstream promoter, creating a conflict between terminating and initiating RNAPII. In this context, the Spt6-Spn1 interaction may act as a gatekeeper of gene boundaries by promoting completion of termination before initiation at adjacent loci. Spt5, another Spt6-interacting factor, is also required for the 3′ transition; however, depletion of Spt5 produces the opposite phenotype with increased RNA signal downstream of pA sites [31]. Notably, the *spt5-QS* mutation suppresses the *spt6-YW* phenotype [8], potentially by restoring the balance of events required for proper termination.

It is difficult to dissect the independent contributions of Spn1 and Spt6 to chromatin organization and transcription, because both associate with elongating RNAPII and function as histone chaperones [32]. Spn1 directly interacts with an N-terminal region of Spt6 [14,33] and recruitment of Spn1 to RNAPII depends on this interaction [34]. Consequently, depletion of Spt6 also reduces Spn1 association with RNAPII, as observed in *spt6-1004* mutants [8]. Dissecting Spt6 functions that are independent of Spn1 therefore requires domain-specific perturbations that preserve the Spn1-interaction region [24]. Conversely, Spn1–Spt6 interaction mutants reduce the amount of Spn1, but not Spt6, associated with RNAPII [8], providing a means to investigate the consequences of reduced Spn1 recruitment while maintaining Spt6 associations with RNAPII.

Loss of Spt6 and Spn1 from RNAPII in *spt6-1004* mutants causes increased antisense transcription, spurious intragenic RNAPII initiation, and widespread changes in nucleosome organization [8,12]. Similar effects occur in Spn1–Spt6 interaction mutants, but generally to a lesser extent [8,15,34]. The present analysis shows that nucleosome organization at the TSS is severely disrupted in *spt6-1004* mutants but only modestly affected in several Spn1–Spt6 interaction mutants, whereas the effects at the pA sites are more comparable. This supports a particularly important role for Spn1 in chromatin organization at gene ends. Consistent with the stronger TSS phenotype, transcription is globally repressed in *spt6-1004* mutants, in line with the requirement for Spt6 to overcome the +1 nucleosome barrier and promote escape from RNAPII promoter-proximal pausing [7,35]. In the Spn1–Spt6 interaction mutant *spt6-YW*, by contrast, transcriptional effects are strongest at genes in tandem orientation, which is more consistent with defective termination of the upstream gene than with a general defect in transcription initiation. Together, these results distinguish an Spn1-sensitive role at 3′ gene ends from broader Spt6-dependent functions in transcription initiation and elongation.

## 4. Materials and Methods

### 4.1. Public Sequencing Datasets Used in This Study

Previously published Saccharomyces cerevisiae MNase-seq, NET-seq, TSS-seq and ChIP-seq datasets were reanalyzed to investigate the effects of impaired Spt6-Spn1 interaction on nucleosome organization and transcription. MNase-seq and RNAPII ChIP-seq datasets comprising WT, *spn1-R263D*, *spn1-F267E*, and *spt6-F249K* strains were obtained from [15] (GEO accession GSE284959). MNase-seq, NET-seq, and TSS-seq datasets comprising WT, *spn1-K192N*, *spt6-YW* and *spt6-YW spt5-QS* strains were obtained from [8] (GEO accession GSE160821). MNase-seq and TSS-seq datasets comprising WT and *spt6-1004* strains were obtained from [12] (GEO accession GSE115775). ChIP-exo datasets for CTDSer5, CTDSer2, Spn1, and Spt6 were obtained from [21] (GEO accession number GSE147927).

### 4.2. Reference Genome and Gene Annotation

All analyses were performed using the Saccharomyces cerevisiae R64-1-1 genome assembly (sacCer3). The genome sequence and gene annotation were obtained from Ensembl release 113. Analyses were restricted to protein-coding genes. Genes overlapping a custom blacklist containing mitochondrial DNA, the ribosomal DNA locus, the TRP1 gene, and additional regions with irregular sequencing profiles were excluded. Ribosomal genes were additionally removed, and only genes classified as “Verified” in the Saccharomyces Genome Database (SGD) were retained, resulting in 5069 protein-coding genes.

### 4.3. MNase-Seq Data Analysis

MNase-seq data were reanalyzed from raw sequencing data using the nucDetective pipeline (v1.2) [16]. The nucDetective Profiler workflow was run to generate normalized nucleosome profiles and to check consistency of biological replicates and MNase digestions using default parameters and specifying an effective genome size of 12,157,105 bp. Replicate 4 of the WT samples at 37 °C taken from [8] was excluded from downstream analysis because its nucleosome profile was inconsistent with those of the other biological replicates. During quality control, the labels of *spn1-R263D* replicate 1 and *spn1-F267E* replicate 1 were found to have been exchanged and were corrected accordingly.

Downstream nucleosome features, including nucleosome positioning, fuzziness and array regularity, were further analyzed using the nucDetective Inspector workflow. Quantile normalization of nucleosome profiles was disabled (--normalize_profiles false), and whole-genome nucleosome regularity analysis was performed using a nucleosome repeat length of 155 bp (--regularity_ref false, --avgNRL 155).

### 4.4. Definition of +1 and T-1 Nucleosomes

+1 and T-1 reference nucleosomes were defined using the WT MNase-seq samples from [15]. Nucleosome positions identified by nucDetective were centered to their dyad positions. For each gene, the nucleosome dyad closest to the annotated TSS or pA site was determined independently in each replicate. Nucleosomes located more than 75 bp from the TSS or pA site were excluded. When a proximal nucleosome was identified in more than one WT replicate, the nucleosome with the highest occupancy score was selected. Using these criteria, no proximal nucleosome was identified for 281 TSSs and 475 pA sites. In these cases, the annotated 5′ or 3′ end of the gene, respectively, was used as the reference position.

### 4.5. Sorting by Nucleosome Regularity

Local nucleosome array regularity was quantified using the whole-genome power spectral density (PSD) regularity profiles generated by the nucDetective Inspector workflow from the WT MNase-seq samples taken from [15]. deepTools (v3.5.4) computeMatrix was used to calculate the sum of the regularity signal within a 300 bp interval downstream of the +1 or upstream of the T-1 reference nucleosomes [36]. The median value across the three WT replicates was assigned to each gene as its nucleosome array regularity score.

### 4.6. Classification of Gene Orientation

Gene orientation was determined separately relative to the +1 and T-1 nucleosomes using the nearest verified protein-coding gene. At the T-1 nucleosome, genes with a neighboring gene within 500 bp downstream were classified as tandem when the neighboring gene was transcribed in the same direction and as convergent when it was transcribed in the opposite direction. Genes without another verified protein-coding gene within 500 bp were classified as isolated.

At the +1 nucleosome, genes were classified as tandem when the nearest upstream gene within 500 bp was on the same strand and as divergent when it was on the opposite strand. Genes without a neighboring verified protein-coding gene within 500 bp were classified as isolated.

### 4.7. Nucleosome Repeat Length Estimation

Nucleosome repeat length (NRL) was estimated from aggregate nucleosome occupancy profiles downstream of the +1 or upstream of the T-1 nucleosomes. Genes were grouped by gene orientation and nucleosome array regularity quartile. Meta-nucleosome occupancy profiles were smoothed using LOESS with a span of 0.1, and nucleosome positions were defined as local maxima upstream of the T-1 or downstream of +1 nucleosome. The NRL was calculated as the slope of a linear regression of the first four consecutive nucleosome positions against their ordinal positions. Estimates with NRL < 150 bp or linear regression fits with R^2^ < 0.99 were excluded.

### 4.8. NET-Seq Analysis and Quantification of Termination-Proximal Stalling

Strand-specific and library-size normalized NET-seq profiles were obtained directly from GEO (accession GSE160821) [8]. Profiles from two biological replicates were merged, and the signal in the sense direction of the underlying gene was quantified in 10 bp bins. The analysis was restricted to genes with at least 1500 bp length. Termination proximal stalling was assessed as the median NET-seq signal in the gene body taken from 1250 to 750 bp upstream of the T-1 nucleosome, whereas the pA-proximal signal was defined as the median signal from −100 to +100 bp around the T-1 nucleosome. A pseudocount of 0.01 was added. Termination proximal stalling was calculated as the log2 ratio of the pA-proximal NET-seq signal to the gene body signal.

### 4.9. TSS-Seq Analysis

Strand-specific TSS-seq profiles were obtained directly from GEO (accessions GSE160821 (library-size normalized) and GSE115775 (spike-in normalized)) [8,12]. The TSS-seq data were analyzed analogously to the NET-seq data described above, except that a pseudocount of 0.5 was used instead of 0.01.

### 4.10. ChIP-Exo Data Analysis

ChIP-exo datasets were analyzed as described in [37] starting from raw sequencing files obtained from GEO (accession number GSE147927) [21]. For factor occupancy correlation analysis with nucleosome array regularity, the normalized ChIP-exo signal was quantified within −250 to +250 bp of the T-1 nucleosome position. Factor occupancy and nucleosome-regularity scores were log2-transformed. Genes with log2 regularity scores ≤ 15 or nonfinite occupancy values were excluded. Linear regression was performed separately for convergent, tandem, and isolated genes, and observations with Cook’s distance > 0.01 were excluded before fitting the final model.

## Data Availability

The data presented in this study are available in Gene Expression Omnibus (GEO) at [https://www.ncbi.nlm.nih.gov/geo/] (accessed on 19 August 2026). Accession numbers for each dataset are stated in Section 4 in paragraph “Public sequencing datasets used in this study”. The nucDetective pipeline (v1.2) used in this paper is available at GitHub (https://github.com/uschwartz/nucDetective, accessed on 19 August 2026). The code used to analyze the data generated in this study is available at GitHub (https://github.com/uschwartz/SPN1_nucleosomes_termination, accessed on 27 August 2026).

## Supplementary Materials

The following supporting information can be downloaded at https://www.mdpi.com/article/10.3390/epigenomes10040061/s1: Figure S1: Characterization of Spn1-Spt6 interaction mutants; Figure S2: Gene length distributions across nucleosome array regularity quartiles; Figure S3: Nucleosome occupancy in Spn1-Spt6 interaction mutants and *spt6-1004*; Figure S4: Occupancy of RNAPII, Spn1, and Spt6 at pA sites is associated with nucleosome array regularity; Figure S5: *spt6-1004* affects transcription initiation broadly; Supplementary Table S1: Reference Annotations.

## Abbreviations

The following abbreviations are used in this manuscript:

bp	Base pairs
CTDSer2	Ser2-phosphorylated C-terminal domain of RNAPII
GEO	Gene Expression Omnibus
kb	Kilobases
LOESS	Locally estimated scatterplot smoothing
NRL	Nucleosome repeat length
pA	Polyadenylation
PSD	Power spectral density
RNAPII	RNA polymerase II
T-1	Termination -1 nucleosome
TSS	Transcription Start Sites
WT	Wild type
