# Supplementary material for: Spn1 Maintains Nucleosome Organization at Transcription End Sites
Source: Epigenomes. 2026 Sep 30;10(4):61. doi: 10.3390/epigenomes10040061 (PMC13692419; doi:10.3390/epigenomes10040061)
Supplement: Supplementary file 1 [file epigenomes-10-00061-s001.zip › Supplementary Materials.pdf]

Supplementary Materials for

Spn1 maintains nucleosome organization at transcription end sites

Uwe Schwartz

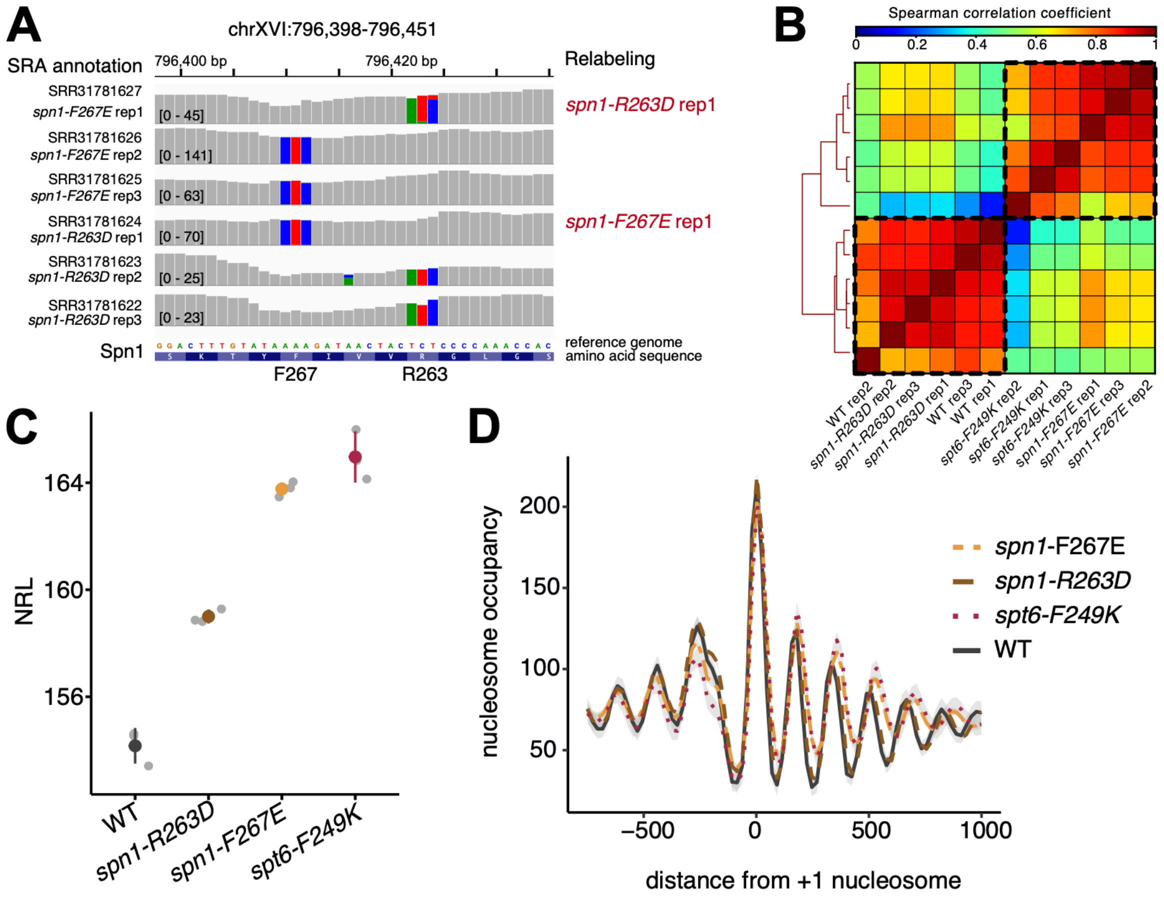

Figure S1. Characterization of Spn1-Spt6 interaction mutants

- **(A)** Genome browser view of the Spn1 locus showing MNase-seq read alignment for the Spn1-Spt6 interaction mutants *spn1-R263D* and *spn1-F267E* taken from (Chen *et al.*, 2025). The nucleotide sequence of the reference genome and the Spn1 amino acid sequence are shown below. The positions of the mutated amino acids F267 and R263 are indicated. Histograms show nucleotide coverage, whereas colored bars (blue = cytosine, red= thymine, green=adenine) indicate mismatches to the reference genome. Short Read Archive (SRA) accession numbers and corresponding meta-data taken from SRA are indicated on the left. Relabeling of samples, based on the mutations detected in the sequencing reads is indicated on the right.
- **(B)** Sample-to-sample similarity of genome-wide nucleosome positions assessed with nucDetective pipeline, demonstrating high reproducibility among biological replicates and distinct clustering by genotype. Color code for Spearman correlation coefficient is indicated at the top. Dashed boxes highlight the two major clusters: 1) WT and *spn1-R263D* samples and 2) *spn1-F267E* and *spt6-F249K* samples.
- **(C)** Genome wide nucleosome repeat length (NRL) estimates for WT, *spn1-R263D*, *spn1-F267E* and *spt6-F249K* mutant strains assessed using the nucDetective pipeline. Gray dots represent NRL estimates of individual replicates and colored dots indicate the mean NRL across replicates of the indicated genotype. The standard deviation from the mean is indicated as error bar.
- **(D)** Average nucleosome occupancy profiles aligned to the +1 nucleosome. Profiles were calculated using locally weighted scatterplot smoothing (LOESS) across replicates. The gray ribbon indicates the standard error of the LOESS fit.

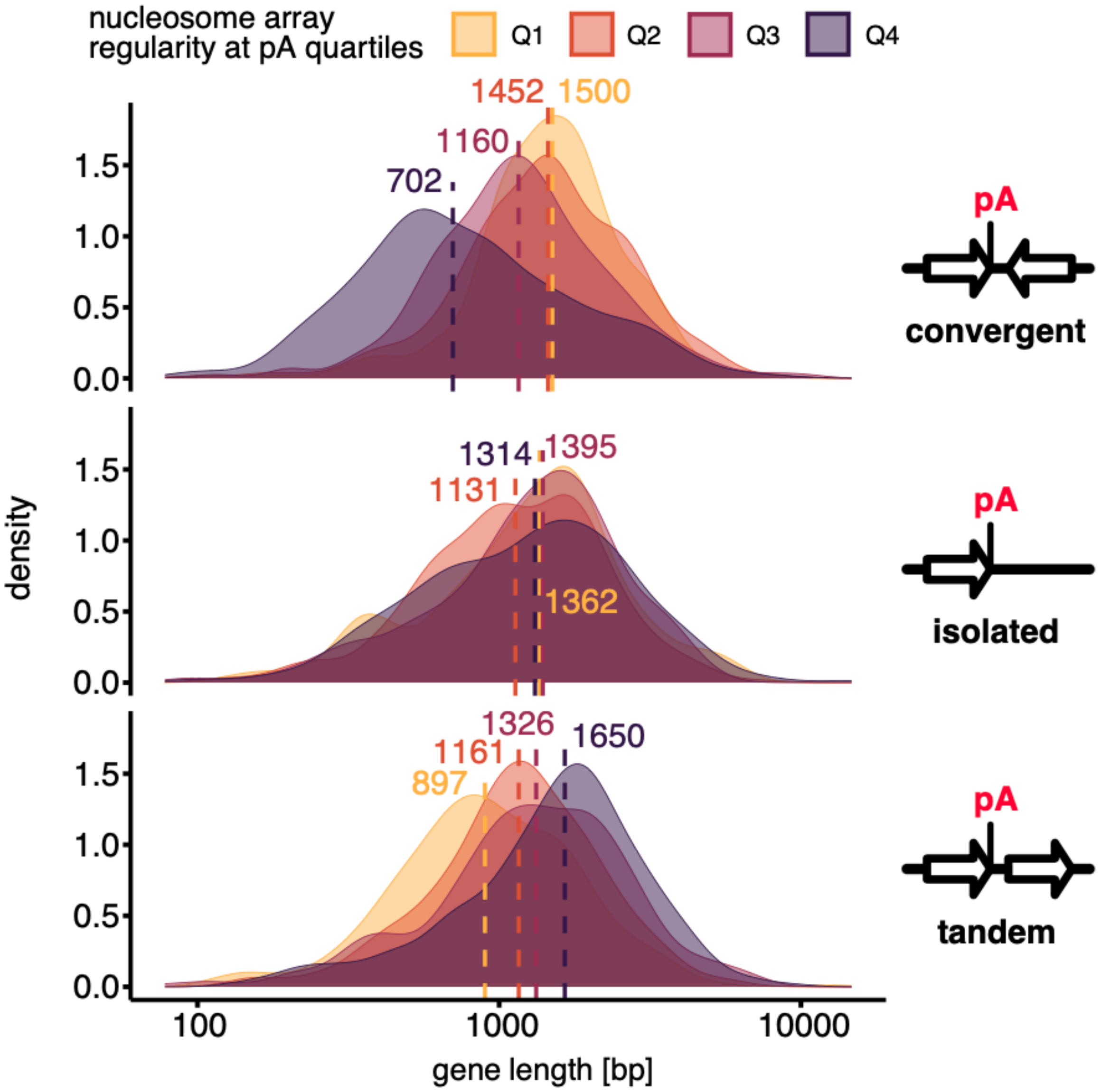

Figure S2. Gene length distributions across nucleosome array regularity quartiles

Gene length distributions of convergent, isolated and tandem genes grouped by nucleosome array regularity at the pA site (quartiles Q1-Q4). Dashed vertical lines and labels indicate the median gene length for each quartile within each gene arrangement class.

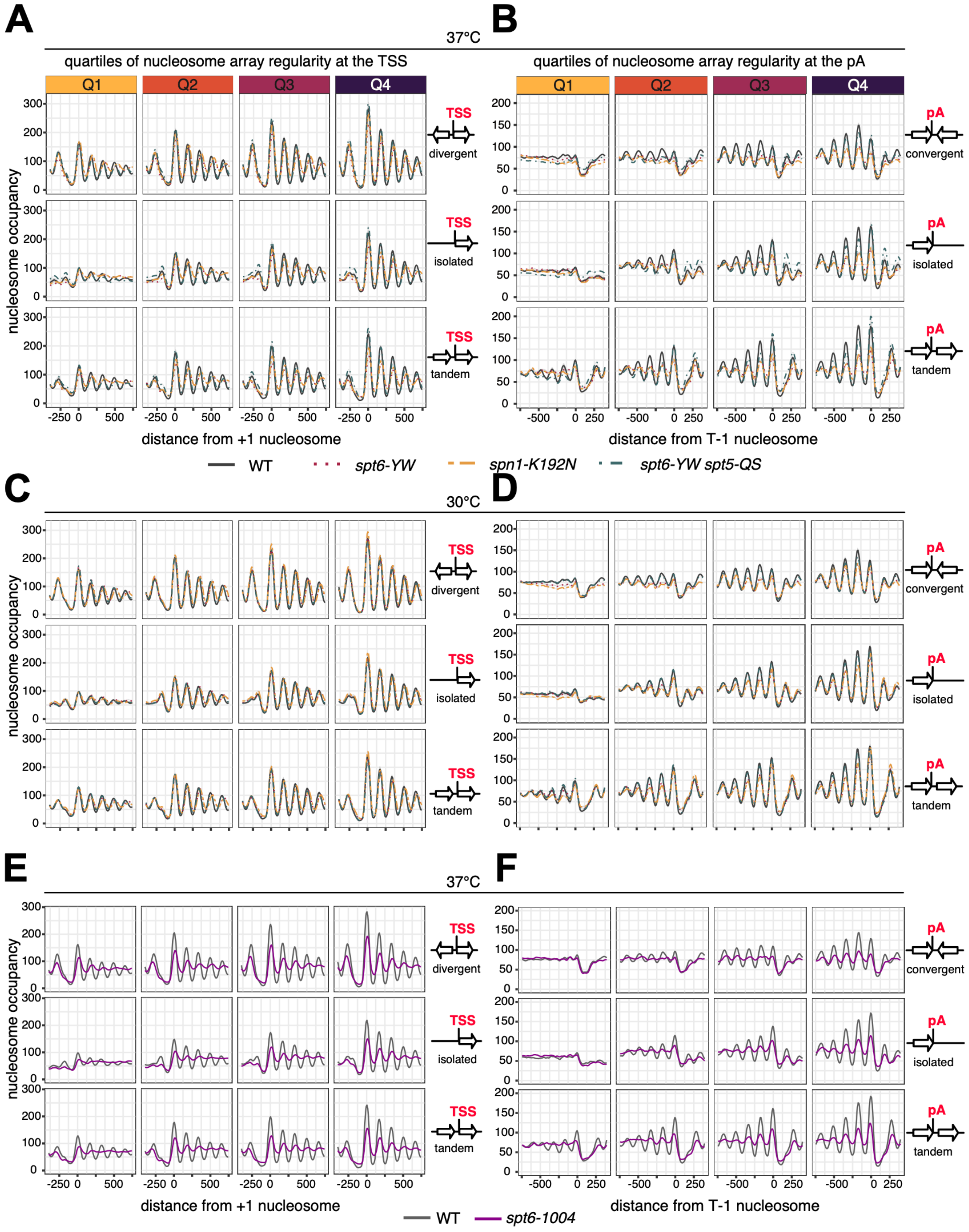

Figure S3. Nucleosome occupancy in Spn1-Spt6 interaction mutants and *spt6-1004*

(A) Average nucleosome occupancy profiles centered on the +1 nucleosome downstream of the TSS. Nucleosome occupancy in WT, *spn1-K192N, spt6-YW* and *spt6-YW spt5-QS* strains at 37°C is shown separately for divergent, isolated and tandem genes and stratified into quartiles (Q1-Q4) according to nucleosome array regularity score at the TSS.

(B) Average nucleosome occupancy profiles centered on the T-1 nucleosome upstream of the pA site. Nucleosome occupancy in WT, *spn1-K192N, spt6-YW* and *spt6-YW spt5-QS* strains at 37°C is

shown separately for convergent, isolated, and tandem genes and stratified into quartiles (Q1-Q4) according to the nucleosome array regularity score at the pA site.

(C) As in (A), but for cells grown at 30°C.

(D) As in (B), but for cells grown at 30°C.

(E) and **(F)** As in (A) and (B), respectively, but showing the *spt6-1004* strain at 37°C.

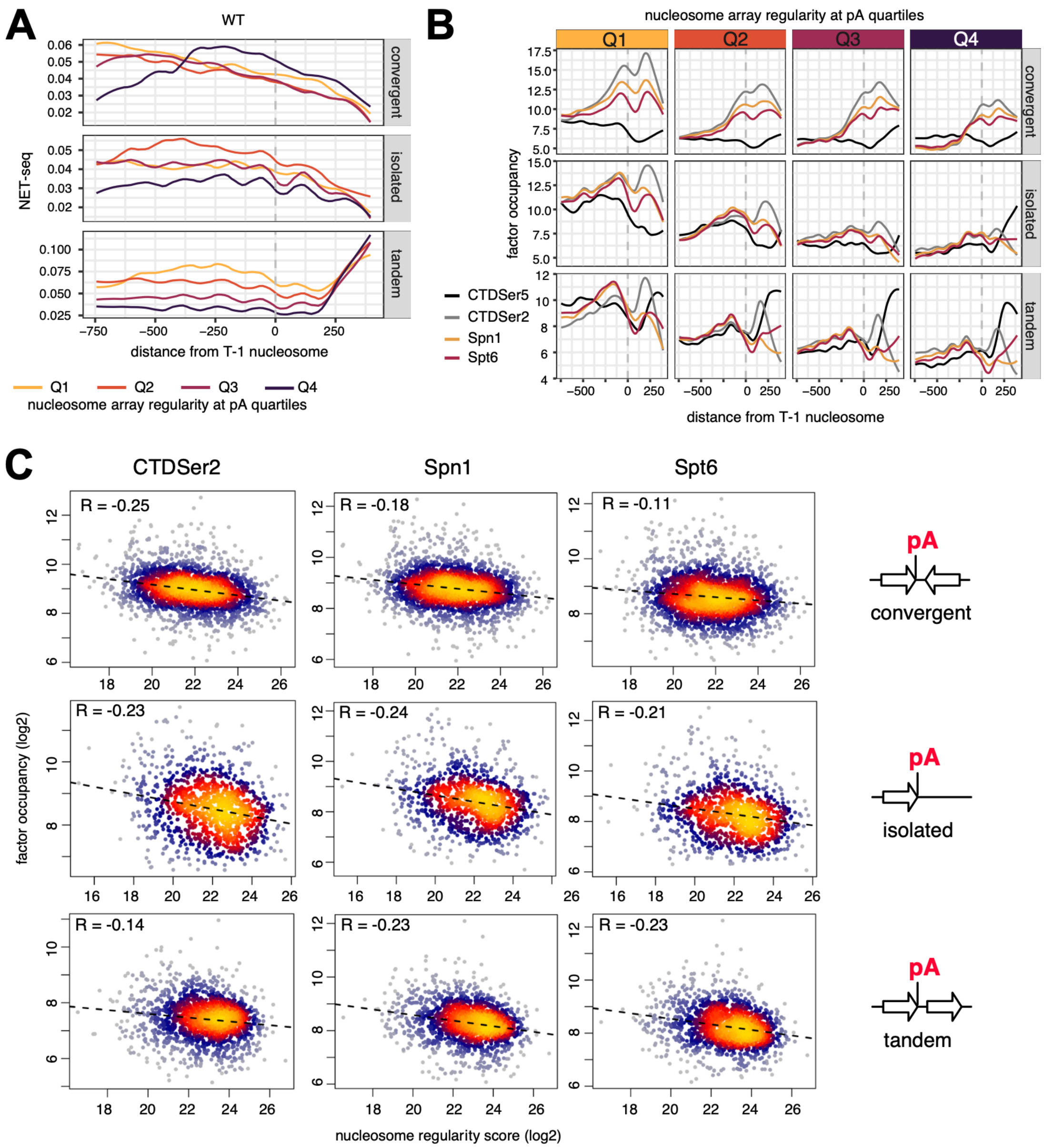

Figure S4. Occupancy of RNAPII, Spn1, and Spt6 at pA sites is associated with nucleosome array regularity

- **(A)** Strand specific NET-seq profiles of transcriptionally engaged RNAPII centered on the T-1 nucleosome in WT cells at 37°C. Profiles are shown separately for convergent, isolated, and tandem genes and were stratified into quartiles (Q1-Q4) according to nucleosome array regularity at the pA site.
- **(B)** ChIP-exo occupancy profiles of initiating RNAPII (Ser5 phosphorylation of the C-terminal domain; CTDSer5), elongating RNAPII (Ser2 phosphorylation of the C-terminal domain; CTDSer2), Spn1 and Spt6 centered on the T-1 nucleosome. Genes are grouped as convergent, isolated, or tandem arrangements and stratified into quartiles (Q1-Q4) according to nucleosome array regularity at the pA site.
- **(C)** Correlation between nucleosome array regularity and CTDSer2, CTDSer5, Spn1, and Spt6 occupancy at the pA site in WT cells. Factor occupancy was quantified as the summed of the ChIP-exo signal within −250 to +250 bp of the T-1 nucleosome . Dashed lines indicate linear regression fits, and the Spearman correlation coefficients (R) are shown in each panel.

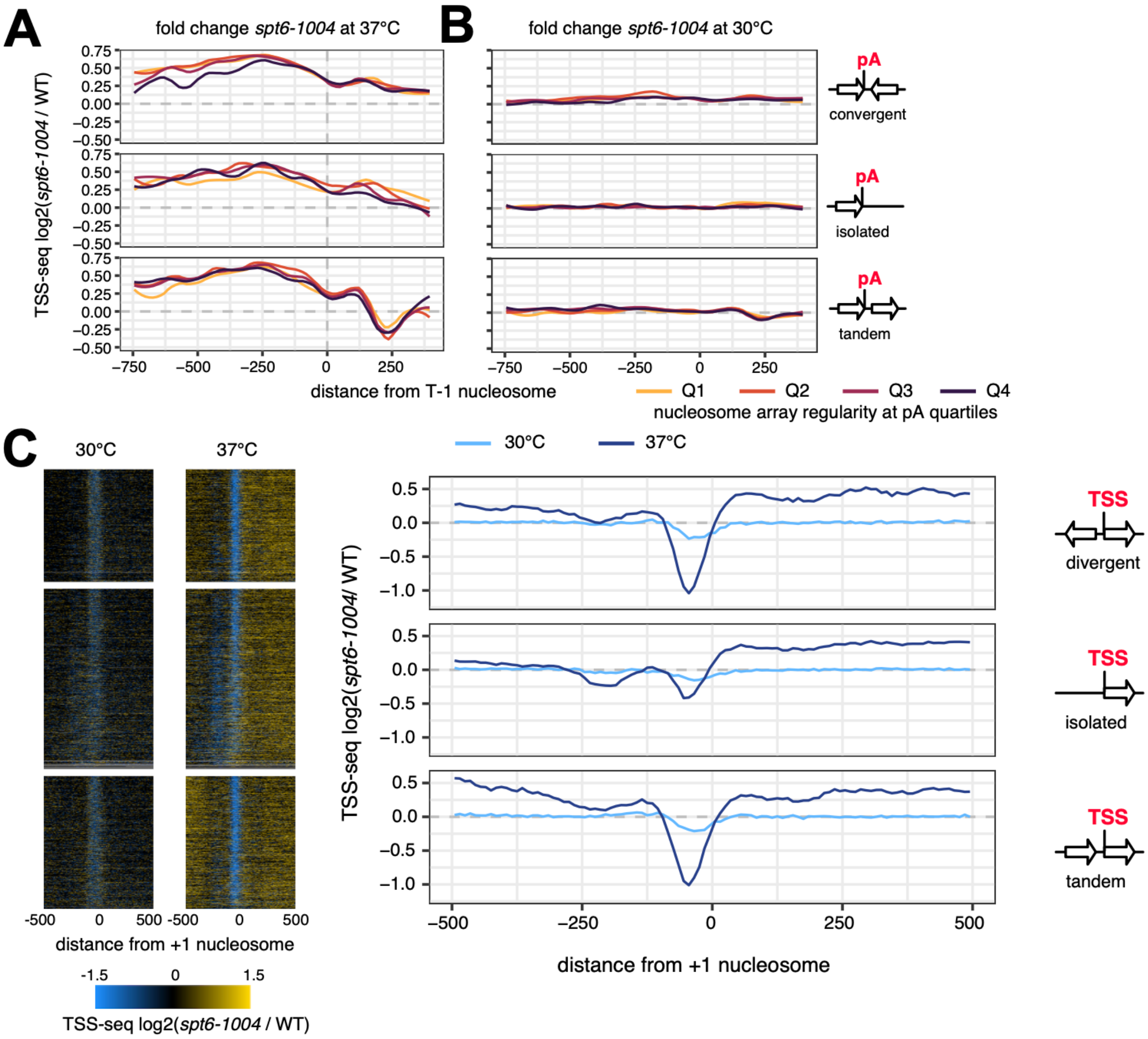

Figure S5. *spt6-1004* affects transcription initiation broadly

(A) Spike-in normalized log2 fold changes in TSS-seq signal in *spt6-1004* cells relative to WT at 37°C. Meta-gene profiles show the average for convergent, isolated, and tandem genes and are stratified into quartiles (Q1-Q4) according to nucleosome array regularity at the pA site.

(B) As in **(A)**, except that log2 fold-changes are shown at the permissive temperature of 30°C.

(C) Changes in transcription initiation centered on the +1 nucleosome in *spt6-1004* relative to WT. Heatmaps show log2 fold changes at permissive and nonpermissive temperatures, as indicated. Corresponding, meta-gene profiles are shown on the right. Genes were grouped as divergent, isolated, or tandem.
